# “Addressing the impact of stroke risk factors in a case control study in tertiary care hospitals”: a case control study in Tertiary Care Hospitals of Peshawar, Khyber Phukhtoonkhwa (KPK) Pakistan

**DOI:** 10.1186/1756-0500-6-268

**Published:** 2013-07-12

**Authors:** Syed Muhammad Adnan Shah, Syed Muhammad Salman Shah, Saima Khan, Shahzad Ur Rehman, Zakir Ahmad Khan, Wisal Ahmed, Zubair

**Affiliations:** 1 Ayub Medical College, Abbottabad, K.P.K, Pakistan; 2 Khyber Medical College, Peshawar, K.P.K, Pakistan; 3 Khyber Girls Campus, Peshawar, K.P.K, Pakistan

**Keywords:** Stroke, Hypertension, Smoking, Diabetes, Sedentary lifestyle, Diet, B.M.I

## Abstract

**Background:**

Stroke was the second most common cause of death worldwide in 2004, resulting in 5.7 million deaths. This case controlled study was conducted in Tertiary care hospitals of Peshawar, KPK Pakistan about common risk factors of stroke and the impact of these risk factors.

**Method:**

Study was done at Government Tertiary Care Hospitals of Peshawar namely Khyber Teaching Hospital (KTH), Lady Reading Hospital (LRH) and Hayatabad Medical Complex (HMC).The hospital based case–control study included 50 cases (stroke patients) and 100 controls (non-stroke patients). This study was accomplished from 24th April 2012 to October 2012 in tertiary care hospitals of Peshawar. A proper questionnaire was used to collect data from all the cases and controls, which was recorded in the form of tables and graphs. The risk factors studied were Hypertension, Smoking, diabetes, sedentary lifestyle, cardiac problems, B.M.I, diet, stress and family history of stroke. Anthropometric (weight, height, body mass index) measurements were done on all patients. For determination of association and impact of these risk factors, analyses were performed by calculation of Chi-Square test and confidence interval using SPSS version 16 program.

**Results:**

Comparing the cases with controls, hypertension (p = 0.000), sedentary life style (p = 0.000), cardiac problems (p = 0.009), diabetes mellitus (p = 0.010), smoking (p = 0. 042) were significant risk factors whereas B.M.I (p = 0. 393), stress (p = 0.705), family history of stroke (p = 0.729), diet (p = 0.908), were not found to be statistically significant risk factors. The most significant risk factor was systemic hypertension (OR = 4.16) followed by sedentary life style (OR = 3.60), cardiac problems (OR = 2.74) diabetes (OR = 2.49) and smoking (OR = 2.05).

**Conclusion:**

Hypertension, Smoking, diabetes, sedentary lifestyle and cardiac problems have strong correlations and association with Stroke and are the major risk factors of stroke. Prevention of these risk factors can lead to decrease in the incidence of stroke. B.M.I, diet, stress and family history of stroke had no significant association.

## Background

Stroke also known as cerebrovascular accident, is the rapid loss of brain function due to a disturbance in the blood supply to the brain. This can be due to ischemia caused by thrombosis, arterial embolism, or a hemorrhage [1]. As a result, the affected area of the brain cannot function, which might result in hemiparesis or hemiplegia, an inability to understand or formulate speech (aphasia), or hemianopia [2].

A stroke is a medical emergency and can cause permanent neurological damage and death. Risk factors for stroke include old age, hypertension, prior stroke or transient ischemic attack (TIA), diabetes, hyperlipidemia, tobacco smoking and atrial fibrillation [2]. The most important modifiable risk factors for stroke are hypertension and atrial fibrillation, (although the magnitude of this effect is small: the evidence from the Medical Research Council trialssuggests that 833 patients have to be treated for one year to prevent one stroke) [3,4]. Stroke is the second leading cause of death worldwide and the leading cause of functional impairments in adults, which affects both patients and their relatives in most regions [5]. WHO defines stroke as rapidly developed clinical signs of focal or global disturbance of cerebral functions lasting more than 24 hours or leading to death with no apparent cause other than a vascular origin [6]. The two main types of stroke include ischemic stroke and hemorrhagic stroke. Ischemic stroke accounts for about 75% of all strokes and occurs when a blood clot, or thrombus, forms that block blood flow to part of the brain. If a blood clot forms somewhere in the body and breaks off to become free-floating, it is called an embolus. This wandering clot may be carried through the bloodstream to the brain where it can cause an ischemic event. Conversely, hemorrhagic stroke occurs when a blood vessel on the brain’s surface ruptures and fills the space between the brain and skull with blood (subarachnoid hemorrhage) or when a defective artery in the brain bursts and fills the surrounding tissue with blood (cerebral hemorrhage). Both result in a lack of blood flow to the brain and a buildup of blood that exerts pressure on the brain [7].

The outcome after a stroke depends on where the stroke occurs and how much of the brain is permanently affected. Smaller strokes may result in minor problems, such as weakness in an arm or leg. Larger strokes may lead to paralysis or even death. Many stroke patients are left with weakness on one side of the body, difficulty speaking, incontinence, or bladder problems [7]. Prospective studies have revealed different risk factors for the various stroke sub types [8]. The risk factors for Ischemic stroke include aging, hypertension, diabetes, smoking, history of cardiovascular diseases, atrial fibrillation, and left ventricular hypertrophy [8,9]. In 2002, the number of deaths due to stroke reached 5.54 million worldwide. Though the incidence is falling in Europe, it is rising in South Asia. According to the 2002 survey, 20% of the 5.54 million deaths due to stroke occurred in South Asia (Sri Lanka, India, Pakistan, and Bangladesh). In KPK, one study reported a prevalence of 4.8% in both men and women. This is the highest ever prevalence of stroke in the world. The INTERSTROKE study, involving 22 countries, demonstrated that ten risk factors account for 90% of the population attributable risks for stroke events. However, not all potential risk factors were considered in the INTERSTROKE study [10]. The INTERHEART case–control study reported that hypertension was especially responsible for stroke - Other causes included smoking, diabetes, hyperlipidemia, low-fiber diet, obesity, sedentary lifestyle, stress, cardiac abnormalities (e.g., atrial fibrillation), previous history of stroke, and various non-modifiable causes (e.g., age, sex, etc.).

There is a paucity of reliable information on the possible risk factors for stroke in the developing countries [11]. A better understanding of the relative importance of the risk factors could lead to better secondary prevention and thus limit the future stroke burden in the increasingly elderly population. Thus, in this hospital-based case–control study we aimed to establish the association and to determine the known and emerging risk factors of stroke and to assess the contribution of these risk factors to the causation of stroke.

## Methods

### Study design

The study design for our project was a hospital based control study.

#### Definition of case control study

Case control study is an analytical study in which past history of exposure suspected risk factors are compared between cases and controls.

This study design was selected because it is rapid, inexpensive and easy to carry out. This study also allows identification of several etiological agents.

### Study setting

We selected three government tertiary care hospitals of Peshawar KPK, LRH and HMC. All these hospitals have developed recent advances in health and education and now considered as top government hospitals in KPK. In addition, they also provide teaching services to medical students.

### Study duration

The duration of our study was from 24th April 2012 to October 2012.

#### Sample size

The sample size was 150 people, containing fifty cases and hundred controls.

#### Study limitations

During this study following problems were encountered.

1 Difficulty in selecting proper control group.

2 Non availability of different diagnostic modalities.

3 Non-cooperative patients.

4 We had to rely on the patient’s memory and hence the possibility of recall bias.

#### Ethics

The sample of 150 subjects was considered to be sufficient for this study, which adhered to the principles of the Declaration of Helsinki, and was approved by independent ethics committees of LRH, KTH and HMC. We obtained written informed consent in all cases to participate in the study.

### Data collection procedure

We use a proper questionnaire to collect data from all the cases and controls, which were recorded in the form of tables and graphs. For the determination of association and impact of these risk factors, analysis was performed using calculation of odd ratio (OR) and confidence internal (CI) using SPSS program. Various clinical examinations were done as;

Calculating body mass index [12]:

BMI = WEIGHT (KG)/HEIGHT * 2 (m * 2).

BMI categories

● UNDERWEIGHT = < 18.5

● NORMAL WEIGHT = 18.5 – 24.9

● OVER WEIGHT = 25–29.9

● OBESITY = BMI OF 30 OR GREATER

A) Weight

The weight was measured in the following way

➣ The scale was set at zero reading.

➣ The patient removed his shoes and heavy outer clothing (jackets, sweaters, hats, and vest) and empty pockets to the possible extent.

➣ The patient stepped on the platform, facing away from the scale readout, with both feet on the platform and remained still with arms hanging naturally at the side and looking forward.

➣ We read the weight valve to the nearest to ¼ or 0.1 kg.

➣ We had the patient steps of the scale and take a second measurement repeating the steps above. (Measurements should agree within 0.1 kg or ¼ pound, if not measured again until this standard is met).

➣ If using a balance beam scale return the weight to zero position.

B) Height

➣ The patient has to remove shoes, hats and hair ornaments to the extent possible.

➣ The patient stood on the footplate or uncarpeted floor with back against the stadiometer rule.

➣ The patient's legs should be together. (In contact at some point whatever touches first.)

➣ We assured patients' legs were straight; arms were at the sides, and shoulders were relaxed.

➣ We assured the back of the patient’s body touches/had contact with the stadiometer at some point, preferably with heels, buttocks, upper back and head touching the measuring surface.

➣ The patient’s body was in a straight line (mid axillary line parallel to the stadiometer).

➣ We asked the patient to breathe in and hold his or her breath while being measured.

➣ We used Demi-span for calculation of height of bedridden patients. We measured the distance from the middle of the sternal notch to the tip of the middle finger in the coronal plane, height is then calculated from a standard formula http://www.rxkinetics.com/height_estimate.html :

$$\begin{array}{l} \text{Females}\;\text{Height}\;\mathrm{in}\;\left(\mathrm{cm}\right)=\left[1.35*\mathrm{demi}\;\mathrm{span}\;\mathrm{in}\;\left(\mathrm{cm}\right)\right]\\ \;+60.1 \end{array}$$

$$\begin{array}{l} \text{Males}\;\text{Height}\;\mathrm{in}\;\left(\mathrm{cm}\right)=\left[1.40*\mathrm{demi}\;\mathrm{span}\;\mathrm{in}\;\left(\mathrm{cm}\right)\right]\\ \;+57.8 \end{array}$$

Calculating BMI:

After calculating weight and height the is calculated as

$$\mathrm{BMI}=\text{WEIGHT}\;\left(\mathrm{kg}\right)/\mathrm{HEIGHT}\;\left(m*2\right)$$

Clinical examination for checking blood pressure:

➣ For cases, B.P and heart rate were recorded at three points/times; at the time of admission, the morning-after admission and at the time of the interview.

➣ For controls, B.P and heart rate were recorded at interview; only hypertension was defined at two approaches, self-reported history of hypertension or the composite of self-reported hypertension or B.P of higher than 160/90.

➣ The American guidelines state that blood pressure below 120/80 mm of Hg is normal, 120 to 139/80 to 89 mm of Hg is pre hypertension, and readings above prehypertension levelare abnormal. Isolated systolic hypertension is defined as an elevated B.P of >140 mm Hg with a normal (<80 mm of Hg) diastolic pressure.

➣ The European and British guidelines have classified a B.P of less than 120/80 mm of Hg as optimal. 120 to 129/80 to 84 mm is normal. 130 to 139/85 to 89 mm of Hg is high normal, and anything above that is classified as hypertension and is divided into three stages [13].

Stage 1: Systolic 140 to 159 mm Hg and/or diastolic 90 to 99 mm of Hg.

Stage 2: Systolic 160 to 179 mm Hg and/or diastolic 100 to 109 mm Hg.

Stage 3: Systolic 180 mm Hg or higher and/or diastolic 110 mm of Hg or higher.

### Blood sugar level

Blood samples for measuring regular blood sugar, and HB1c were taken from cases (within 72 hrs of admission) and controls (at the time of the interview). These tests are used for assessment of blood-glucose level.

Random blood sugar (RBS) and HBA1c will be used for the assessment of blood sugar level.

### HBA1c

The hemoglobin hbA1C test is used to monitor long term glucose (sugar) control in people with diabetes, while daily blood sugar testing gives a picture of the day to day fluctuations. The hemoglobin hbA1C test offers an overview of how well glucose has been controlled over the past two to three months, because the glucose irreversibly binds to hemoglobin for the life of RBC (about 120 days) [14]. Doctors can use the test to determine the person's average blood sugar levels over that time.

### Fasting blood sugar (FBS)

FBS measure blood sugar after you have not eaten for at least 8 hrs. It is often the first test done to check for pre-diabetes and diabetes [15].

### Random blood sugar (RBS)

RBS measures blood glucose regardless of when someone last eats. Several random measurements are taken throughout the day. The random test is useful because glucose level in healthy people does not vary widely throughout the day. Blood glucose that varies widely may mean a population problem. This test is also called a casual blood glucose test [15] (Table 1).

**Table 1:** Fasting and random sugar level

**Types of tests**	**Normal levels**	**Pre diabetes**	**Type 1 or 2 diabetes**
**Simple blood sugar test**	60----99 mg/dl	100---199 mg/dl	>200 mg/dl
**Fasting blood sugar test**	60-----99 mg/dl	100---125 mg/dl	>126 mg/dl

Diabetic ranges based HBA1C:

● Normal range 4.5 to 7.0%

● Good control 6.4 to 7.7%

● Fair control 7.8 to 8.5%

● Poor control above 8.5%

### Smoking status

It is defined as never, former and current smoker. We defined current smoker as an individual who smoked any tobacco in the past 12 months and included those who had quit within the past year. Former smoker is defined as who had quit more than a year earlier.

### Diet assessment

For assessment of diet questions were asked from cases and controls regarding meat, vegetables and fruit intake in the last week.

### Physical activity

Questions were asked regarding exercise tolerance and extent of usual physical activity. The individual was classified as physically active if they were regularly involved in moderate exercise (brisk walking, cycling or gardening etc.) or strenuous exercise (jogging, football etc.).

### Psychosocial stress

For psychosocial stress, we used a combine measure of general stress at home, workplaces and financial status.

### Analysis

We aimed to determine and address the common potential risk factors and their relative risk for stroke. This case control Study was done at Government Tertiary Care Hospitals of Peshawar (KTH, LRH, and HMC) and involved 50 cases (stroke patients) and 100 controls (non-stroke patients).

This case control study was accomplished from 24th April 2012 to October 2012 in tertiary care hospitals in Peshawar. A proper questionnaire was used to collect data from all the cases and controls, which was recorded in the form of tables and graphs. The data were analyzed using SPSS program version 16 software. Chi square test was used as a test the significance and p value less than 0.05 was considered as significant. Odd ratio and relative risk were calculated, summarized and tabulated in Table 2.

**Table 2:** Risk factors of stroke

**Variable**	**Cases (n = 50)**	**Controls (n = 100)**	**P-value**	**Odd ratio**	**Relative risk**
Exercise	18 (36%)	67 (67%)	.000	3.60	2.32
No Exercise	32 (64%)	33 (33%)			
Smoker	24 (48%)	31 (31%)	.042	2.05	1.5
Nonsmokers	26 (52%)	69 (69%)			
Balanced diet	23 (46%)	47 (47%)	.908	1.04	
Fatty diet	27 (54%)	53 (53%)			
Diabetic	24 (48%)	27 (27%)	0.10	2.49	1.7
Non diabetic	26 (52%)	73 (73%)			
Hypertensive	39 (78%)	46 (46%)	.000	4.16	2.7
Non Hypertensive	11 (22%)	54 (54%)	.705	1.15	
Stress	16 (32%)	29 (29%)			
Normal/Non Stress	34 (68%)	71 (71%)			
**B**.**M**.**I**					
Obese	21 (42%)	31 (31%)	.393	1.71	
Overweight	14 (28%)	31 (31%)			
Normal weight	15 (30%)	38 (38%)			
Family history of stroke	26 (52%)	49 (49%)	.729	1.12	
No family history of stroke	24 (48%)	51 (51%)			
Cardiac Problem	18 (36%)	17 (17%)	.009	2.74	1.8
No cardiac problem	32 (64%)	83 (83%)			

## Results

Following are findings regarding different variables in our study.

### Exercise

Among 50 cases, 36% were exercising and 64% were not exercising and among 100 controls 67% were exercising and 33% were not exercising.

### Smoking

Among 50 cases there were 48% smokers and 52% were non-smokers. Among 100 controls 31% were smokers and 69% were non-smokers.

### Diet

Among 50 cases 46% were using balanced healthy diet and 54% were using unhealthy diet and among 100 controls 47% were using balanced diet and 53% were using unhealthy diet.

### Diabetes

Among 50 cases 52% were non-diabetic and 48% were diabetic and among 100 controls 27% were diabetic and 73% were non-diabetic.

### Hypertension

Among 50 cases 78% were hypertensive and 22% were non-hypertensive and among 10 controls 46% were hypertensive and 54% were not hypertensive.

### Stress

Among 50 cases 68% were not stressed and 32% were stressed and among 100 controls 71% were not stressed and 29% were stressed.

### Body mass index

Among 50 cases 30% have a normal BMI, 28% were overweight and 42% were obese. Among 100 controls 37% have a normal BMI, 31% were overweight and 31% were obese.

### Family history of stroke

Among 50 cases 52% were having a family history of stroke and 48% were having no family history of stroke. And among controls 49% have a family history of stroke and 51% have no family history of stroke.

### Cardiac problem

Among 50 cases 36% had diagnosed cardiovascular disease and 64% were having no known cardiac problem and among 100 controls 17% had cardiovascular diseases and 83% had no documented cardiac problems.

## Discussion

Consistent with the findings of other studies, this study showed that Hypertension, Smoking, diabetes, sedentary lifestyle and cardiovascular diseases have strong correlations and association with Stroke and are the major risk factors of stroke while B.M.I, Diet, Stress and family history of stroke had no significant association with stroke.

Hypertension is recognized as the most common risk factor of stroke in this study. Out of n = 50 cases, 39 cases (78%) were hypertensive, having p = 0. 000 and Odd ratio of 4.16 with relative risk of 2.7.

Our findings of hypertension replicate the findings of hypertension in a study conducted in Karachi Taj F et al. [16] and also our values regarding Hypertension is in near range of studies [17,18].

A large case–control study evaluating risk factors for stroke was conducted in Beijing China, has shown that 10 risk factors are associated with 90% of the risk of stroke and, of these modifiable risk factors, hypertension is the most important for all stroke sub types and is a particularly dangerous risk factor for intracerebral hemorrhage [10]. This study showed that many strokes can be predicted and that relatively simple measures, such as blood-pressure control, could reduce the burden of disease. It can be modified with generic medications, and it can also be modified at the population level by implementing such policies as those aimed at reducing salt intake, losing weight and encouraging exercise.

According to a study [19] held in department of clinical, cardiovascular & immunological sciences II, Federico university, Naples, Italy, PI(A2) polymorphism is a genetic determinant of ischemic stroke in a selected high risk hypertensive population. They studied 140 genotyped hypertensive control individuals and 28 hypertensive patients with ischemic stroke. The frequency of the PI (A2) allele was higher among stroke versus non-stroke patients (Stroke, 46.4%; non stroke, 22.6% p = 0.01). Multiple regression analysis taking into account this polymorphism among other factors known to contribute to ischemic stroke confirmed the PI (A2) allele as an additive risk factor for stroke, increasing the risk of stroke by 2.9 (95% confidence interval =1.2-6.85, p < 0.02).

According to another study [20] held at department of medicine, cardiovascular and immunological sciences, Federico II university of Naples, Naples, Italy, expression of calcium/calmodulin-dependent kinase IV plays a pivotal role in blood pressure regulation through control of endothelial nitric oxide synthase activity. They characterized in this study, the cardiovascular phenotype of CaMK4(−/−) mice and they displayed a typical hypertensive phenotype, including high blood pressure levels, left ventricular hypertrophy, vascular and kidney damage, and reduced tolerance to chronic ischemia and myocardial infarction compared with wild-type littermates. In population study they found that the rs10491334 variant associates with a reduction in the expression levels of CaMKIV in lymphocytes from hypertensive patients. Their results provide evidence that CaMKIV plays a pivotal role in blood pressure regulation throughthe control of endothelial nitric oxide synthase activity.

Hypertension is the single most important modifiable risk factors for both ischemic and hemorrhagic strokes. The National Health Survey of Pakistan (1990–4) highlighted the enormous burden of hypertension in the country. Twenty-two percent of the urban Pakistani population over the age of 15 years, and a third of those aged 45 years and above, had hypertension [21]. The overall prevalence of hypertension in Pakistanis aged 15 years and above was 19.0% (95% CI; 18.9-19.1) [22].

Both ischemic and hemorrhagic stroke has strong gradients with blood pressure. For each rise of 20 mm of Hg in systolic blood pressure, the relative risk of ischemic and hemorrhagic stroke increases 2.23 and 3.18 times, respectively. Fall in blood pressure observed over the 20th century may lead to bigger reduction in the incidence of hemorrhagic stroke compared with ischemic stroke [23].

Diabetes mellitus is another important modifiable risk factor for stroke. Our study showed that n = 24, 48% among cases were diabetic having p = 0.010, odd ratio of 2.49 and relative risk of 1.7. Our study findings in perspective of Diabetes Mellitus are in near range of studies [24,25].

Pakistan holds more than 5 million diabetic patients which are expected to rise to 3.9 million by 2020, leading Pakistan to 4th most populous country accommodating patients with diabetes mellitus [26]. According to the National Health Survey, 25% of peoples over the age of 45 years in Pakistan suffer from Diabetes mellitus.

Both type 1 and type 2 diabetes increase risk of stroke. In addition to the stroke risk caused by diabetes itself, many diabetics also have high blood pressure, high cholesterol and are overweight, all additional risk factors for stroke. Data from the Framingham Heart Study indicate that diabetes may have a greater impact on stroke risk in older women compared with men [27].

In addition to having increased risk of stroke, people with diabetes who have stroke often fare worse than non-diabetic stroke victims—especially women. One study found that 16% of stroke deaths in men and 33% of women were attributable to diabetes [28].

Diabetes increases risk of stroke because the excess glucose in blood causes vasculopathy, making it more likely to have hypertension and atherosclerosis. In addition, diabetes increases the risk of blood clots, which can cause both heart attack and stroke. The excess sugar in the blood has a direct effect on blood vessel walls, binding with and altering the structure of proteins and molecules that line the blood vessel, making the vessels thicker, less elastic, and more likely to initiate thrombosis. Thicker and less elastic blood vessels means that blood has a harder time flowing through narrower openings, and will have to do so at a higher pressure. These changes cause tissue damage termed end-organ damage. Smaller spaces for blood to flow means a greater likelihood that a clot could completely block a vessel, causing a stroke or heart attack [29].

In type 1 diabetic patients, maintaining blood sugar levels close to a normal value reduces the risk of heart attack, stroke, or death from cardiovascular disease by 57% [30]. Patients with diabetes, controlling high blood pressure are an important step in reducing risk of stroke. A study from the UK found that tight blood pressure control (average blood pressure achieved 144/82 mm Hg) resulted in a 44% lower risk of stroke compared with less stringent control (average blood pressure achieved 154/87 mm Hg). In another study, high blood pressure treatment in elderly diabetic patients resulted in a 20% lower stroke risk [31,32].

In another study [33] held in Acute Stroke unit, university department of medicine and therapeutics, Gardiner institute, western infirmary & faculty of Medicine, University of Glasgow, UK, they Selected 29,500 ischemic stroke patient : 5411 (18.5%) had Diabetes Mellitus, 5019 had a prior stroke (17.1%), and 1,141 (5.5%) had both. They concluded that outcomes from thrombolysis are better than the controls among patients with Diabetes Mellitus, Prior Stroke, or both. They found no statistical justification for the exclusion of these patients from receiving thrombolytic therapy.

According to study of Gaetano Santulli [34], Mishra et al. [35] examined the influence of diabetes mellitus and prior stroke on the outcomes of patients who received thrombolysis vs non-thrombolyzed controls. They found no interaction on outcome between diabetes and prior stroke with thrombolysis treatment. These results conflict with the European Medicines Evaluation Agency’s justification for restricting the use of IV alteplase. Studies have suggested that thrombolysis can be safely used in several groups of patients who do not qualify for treatment due to strict application of exclusion criteria. In addition, most of the commonly cited thrombolytic exclusion criteria are just consensus-based, not evidence-based. It is time to reevaluate the criteria for thrombolysis, adopting a clinical score to stratify the risk, similar to those used in acute coronary syndrome. A good risk assessment tool will be able to identify a gradient of mortality risk by using variables that capture the majority of prognostic information to better evaluate the risk/benefit ratio for each patient [34].

According to statistics from the American Diabetes Association, at least 65% of people with diabetes die from heart disease or stroke, yet more than half don't think they are at risk of stroke [36]. Many studies have shown that having diabetes increases a person’s risk of stroke independently of any other risk factors. It has been estimated that about 40% of all strokes are related to the effects of diabetes [35] and people with diabetes are 2 to 6 times more likely to have a stroke than people without diabetes—the same level of risk associated with already having cardiovascular disease [36].

In this study, we found that smoker among cases were 48%, n = 24 having p = .042, odd ratio of 2.05 with relative risk of 1.5. Our study findings are in the near range of studies [37,38].

Based on the data of the National Health Survey of Pakistan, the overall prevalence of smoking among individuals aged 15 years or older was 15.2%. Gender was the strongest predictor of smoking: the prevalence of smoking was 28.6% in men versus 3.4% in women.

Smoking doubles overall stroke risk compared with nonsmokers [39]. In women younger than 65, smoking is estimated to be responsible for 55% of deaths due to stroke (51% in men) [40]. The Surgeon General's Report on the Health Consequences of Smoking notes that cigarettes with lower yields of tar and nicotine have not been shown to lower risk of heart disease or stroke and should not be considered lower-risk alternatives to regular cigarettes [41]. Smoking approximately doubles a woman's risk of ischemic stroke, the most common type of stroke [41].

Overall, smoking contributes to 12% to 14% of all stroke-related deaths [41]. Among nearly 40,000 US women in the Women's Health Study, those who smoked more than 15 cigarettes a day had a 4-fold increased risk of hemorrhagic stroke caused by sub arachnoid hemorrhage compared with nonsmokers [42,43].

In the ARIC study current smokers were found to have the greatest progression of atherosclerosis over time, at a rate 50% greater than in nonsmokers [44]. Environmental tobacco smoke (ETS) exposure was associated with an approximately 20% greater rate of atherosclerosis progression than for nonsmokers not exposed to ETS. Similar findings have been reported by others [45].

Smoking is believed to induce the development of atherosclerosis by initiating endothelial injury, presumably due to either the production of oxygen radicals or via direct toxic effects of cigarette smoke constituents. Even brief exposure to cigarette smoke has been found to activate leukocytes, stimulating the release of the pro-coagulant, von Willebrand Factor (vWF) and causing endothelial damage [46].

The ramifications of impairment of vasodilation following smoking may place smoking individuals at a higher risk of cerebral ischemic events in two ways. First, due to both a reduced ability to respond to alteration in perfusion pressure, and second, due to lack of vasodilation of the arterial wall as a result of endothelial dysfunction. The notion that this lack of vasodilation of the arterial wall may increase the risk of atherosclerotic plaque rupture, leading to emboli and subsequent infarction, was presented by Kool and colleagues [47].

Examination of serum of smokers reveals that smokers have significantly increased fibrinogen and white blood cell counts when compared to nonsmokers [48], giving further credence to the suggestion that smokers are more prone to coagulation and thrombosis than non-smokers. The ratio of tPA to PAI-1 was found to be lowest in smokers, suggesting that smokers have the lowest potential for fibrinolytic activity that may lead to a greater tendency for thrombus among smokers. The apparent close association between tPA and PAI-1 levels suggests that there is most probably mechanisms ensuring a balance of the activator and inhibitor. In smokers it would appear that this balance is not maintained and this may predispose these individuals to pathological conditions such as stroke [48].

In our study we found that 64%, n = 32, p = 0. 000 having odd ratio 3.60 with relative risk of 2.32 stroke cases have history of sedentary life style.

It has been known for a while that lifelong regular exercise—beginning at around age 15—is associated with up to a 70% reduced risk of ischemic stroke later in life, [49] but there is accumulating evidence that starting exercise in mid-life also helps, Increasing level of activity by 3.5 hours per week can reduce stroke risk by almost 40%, regardless of the age at which the increase is made [50].

Regular physical activity helps reduce risk factors for stroke (and heart disease) including high blood pressure, high cholesterol and triglycerides, diabetes, andobesity. Exercise also keeps improved vasodilation and can reverse or stall the buildup of atherosclerotic plaque in blood vessels, which can lead to clots and stroke [51,52]. Exercise also reduces the likelihood of developing type 2 diabetes—a major risk factor for stroke—by increasing the body's sensitivity to insulin. This in turn makes it easier for your body to maintain suitably low blood sugar levels. Researchers found that any form of exercise reduced stroke risk in all groups, but both higher intensity exercise and longer periods of any exercise translated to a lower stroke risk. Light activity reduced risk by 61%, and moderate-to-heavy exercise reduced risk by 77%. Less than 2 hours of exercise per week was associated with a 58% risk reduction, while exercising for more than 5 hours per week was associated with a 69% risk reduction. "Light-moderate" exercise included walking, calisthenics, dancing, golf, bowling, horseback riding, and gardening. "Heavy" exercise included hiking, tennis, swimming, bicycle riding, jogging, aerobic dancing, handball, racquetball and squash [53].

Compared with “usual” stroke care and recovery, an exercise-intensive program can lead to better outcomes in patient’s endurance, balance, and mobility [54,55].

Our study found that 36%, n = 18 stroke patients were having cardiac problems, having p = 0. 009, odd ratio = 2.74 and relative risk of 1.8. Our values of study were in near range of studies [56].

Heart diseases that are risk factor leading to stroke are Atrial Fibrillation, Left Ventricular Hypertrophy (LVH), Coronary Artery Disease (CAD), Congestive Heart Failure (CHF), Patent Foramen Ovale (PFO) and Valvular heart Diseases. Prompt treatment and prevention can lower the risk of stroke.

AF is responsible for 15% to 20% of all ischemic strokes. It increases the risk of a first stroke 3- to 4-fold [57,58]. The stroke risk is the same whether the AF is persistent or paroxysmal (comes and goes) [59]. In people with AF, the atria's rapid, irregular beat moves blood inefficiently, and the blood inside the left atrial appendage tends to form clots. These clots can break loose and travel through the bloodstream to the brain, where they become lodged in an artery, causing a blocked-vessel (ischemic) stroke [60].

In a study [61] held in Department of Medicine, Faculty of Medicine, University of Colombo, Colombo, Sri Lanka concluded that high prevalence of left ventricular hypertrophy is recognized as a risk factor for stroke . In this study 55 (44 males, 80%) patient with ischemic stroke were studied in the 38 mobilized patients for left ventricular hypertrophy mass index 29, (76.3%) had left ventricular hypertrophy while 19 (50%) had severe hypertrophy, while 25, (65.8%) has concentric hypertrophy.

How LVH predisposes to stroke and other cardiovascular events is still under speculation but multiple mechanisms are proposed. LVH increases the work load of the ventricle and the wall tension by alteration of ventricular geometrics. This is further complicated by increasing myocardial fibrosis that contributes to diastolic dysfunction limiting end diastolic filling capacity and increasing left ventricular end diastolic pressure. These sequences of events are controlled by non-modifiable factors such as genetics and modifiable factors such the renin, angiotensin, aldosterone axis. The increased workload of the ventricles also increases the risk of vascular insufficiency to the inner parts of the hypertrophied myocardium leading to areas of myocardial ischaemia or infarcts. These may create small areas of dyskinetic or hypokinetic myocardium which may serve as areas of origin for small thrombi. However, not all patients have symptoms and some may be undiagnosed till a major event happens [61].

Our study couldn’t find a positive association between Diet, BMI, Stress, Family history and Stroke.

Since most patients in Government tertiary care hospital share similar social and economic background with a rural way of life and agriculture composed mainly of vegetables, cereals, fruits, milk, pulses and meat, making it as a balanced one and is free of hazards of junk and fatty food. Due to these factors, we couldn’t find a positive association between diet and stroke in our study. Since higher BMI is due to excess calorie intake, therefore, most of the factors stated in relation to diet apply to BMI and obesity also.

According to study conducted by Agha khan university Karachi 55.8% cases were found to have a stressful life. Low income, large family size, high dependency ratio, hectic lifestyle are the common determinants of stress. Some of the possible reasons that our study was unsuccessful to find a relation between stress and stroke may be due to increased awareness about family planning have resulted in reduction of family size, resulting in the reduction of the prevalence of stress. People live close to nature away from the hustle and bustle of modern urban life and thus avoid the outcomes of purely materialistic lifestyle like stress, sedentary lifestyle and depression.

According to a study conducted in china by the department of Neurology, Renji Hospital, Family history of hypertension was more frequent in young adults than in middle-aged and very old patients, whereas very old patients were less likely to have a positive family history of hypertension, heart diseases, or stroke. It suggested a genetic component in the pathogenesis of ischemic stroke in young patients.

One of the main reasons that our study couldn’t find an association between a positive family history, and stroke might be because of the fact that most of our cases were in their 50s and 60s and were out of the age group which is more susceptible to the outcomes of genetics regarding stroke.

The most important message from this study, for both clinicians and policymakers, is that adequate preventive measures of modifiable stroke risk factors may contribute to prevention of severe morbidity and mortality.

## Conclusion

In our hospital based case–control study in patients with stroke, hypertension, sedentary lifestyle, cardiac problems, diabetes and cigarette smoking, were significant risk factors. This could be helpful in early identification of subjects at risk for stroke and formulating public health strategy, if proven by a larger population based studies.

## Competing interests

We have no competing interest associated with this paper.

## Authors’ contributions

SMAS: Conception and design of study, generation, collection, assembly, analysis and interpretation of data drafting and revision of the manuscript, approval of the final manuscript. SMSS: Generation collection assembly analysis of data revision of the manuscript and approval of the final version of the manuscript ZAK: Generation, collection, assembly, analysis of data; revision of the manuscript; and approval of the final version of the manuscript. Z.: Conception and design of the study, drafting and revision of the manuscript and approval of the final version of the manuscript. WA: Revision of the manuscript and approval of the final version of the manuscript. SK: Conception and design of the study, drafting and revision of the manuscript; and approval of the final version of the manuscript. SUR: Conception and design of the study, drafting and revision of the manuscript; approval of the final version of the manuscript.
